# Diphtheria And Tetanus Vaccination History Is Associated With Lower Odds of COVID-19 Hospitalization

**DOI:** 10.3389/fimmu.2021.749264

**Published:** 2021-10-07

**Authors:** Jennifer Monereo-Sánchez, Jurjen J. Luykx, Justo Pinzón-Espinosa, Geneviève Richard, Ehsan Motazedi, Lars T. Westlye, Ole A. Andreassen, Dennis van der Meer

**Affiliations:** ^1^ School of Mental Health and Neuroscience, Faculty of Health, Medicine and Life Sciences, Maastricht University, Maastricht, Netherlands; ^2^ Department of Radiology and Nuclear Medicine, Maastricht University Medical Center, Maastricht, Netherlands; ^3^ Department of Psychiatry, Brain Center Rudolf Magnus, University Medical Center Utrecht, Utrecht University, Utrecht, Netherlands; ^4^ Department of Translational Neuroscience, Brain Center Rudolf Magnus, University Medical Center Utrecht, Utrecht University, Utrecht, Netherlands; ^5^ Outpatient Second Opinion Clinic, GGNet Mental Health, Warnsveld, Netherlands; ^6^ Department of Mental Health, Parc Tauli University Hospital, Sabadell, Barcelona, Spain; ^7^ Department of Clinical Psychiatry, School of Medicine, University of Panama, Panama, Panama; ^8^ Department of Medicine, School of Medicine, University of Barcelona, Barcelona, Spain; ^9^ NORMENT, Division of Mental Health and Addiction, Oslo University Hospital & Institute of Clinical Medicine, University of Oslo, Oslo, Norway; ^10^ Department of Psychology, University of Oslo, Oslo, Norway

**Keywords:** COVID-19, vaccination, diphtheria, tetanus, clinical variation

## Abstract

**Background:**

COVID-19 is characterized by strikingly large, mostly unexplained, interindividual variation in symptom severity: while some individuals remain nearly asymptomatic, others suffer from severe respiratory failure. Previous vaccinations for other pathogens, in particular tetanus, may partly explain this variation, possibly by readying the immune system.

**Methods:**

We made use of data on COVID-19 testing from 103,049 participants of the UK Biobank (mean age 71.5 years, 54.2% female), coupled to immunization records of the last ten years. Using logistic regression, covarying for age, sex, respiratory disease diagnosis, and socioeconomic status, we tested whether individuals vaccinated for tetanus, diphtheria or pertussis, differed from individuals that had only received other vaccinations on 1) undergoing a COVID-19 test, 2) being diagnosed with COVID-19, and 3) whether they developed severe COVID-19 symptoms.

**Results:**

We found that individuals with registered diphtheria or tetanus vaccinations are less likely to develop severe COVID-19 than people who had only received other vaccinations (diphtheria odds ratio (OR)=0.47, p-value=5.3*10^-5^; tetanus OR=0.52, p-value=1.2*10^-4^).

**Discussion:**

These results indicate that a history of diphtheria or tetanus vaccinations is associated with less severe manifestations of COVID-19. These vaccinations may protect against severe COVID-19 symptoms by stimulating the immune system. We note the correlational nature of these results, yet the possibility that these vaccinations may influence the severity of COVID-19 warrants follow-up investigations.

## Introduction

The coronavirus disease 19 (COVID-19) pandemic is a global health crisis of unprecedented proportions, having caused millions of deaths (1), as well as severely disrupting everyday life and crippling the global economy (2). While large scale vaccination campaigns are now being implemented, millions will not have access to the vaccines in the near future (3). It is likely that the underlying virus (SARS-CoV-2), in its current or mutated forms, will remain to significantly influence our lives for the foreseeable future. It is therefore essential that we identify factors that moderate its pathogenic effects.

A particularly striking characteristic of the COVID-19 clinical picture is the considerable interindividual variation. Although estimates vary widely, it is evident that a large proportion of individuals infected with SARS-CoV-2 remain asymptomatic or show only mild flu-like symptoms (4), while others develop severe respiratory difficulties that may lead to death (5, 6). Old age and pre-existing health conditions are strong predictors of poor outcomes, as are certain ethnic backgrounds and male sex (7, 8), yet a sizeable portion of apparently healthy individuals also suffer severe outcomes. The cause of this variation remains largely unknown, although differences in immune system function are a likely suspect.

One hypothesis, put forth by several researchers independently, is that previous vaccinations for other pathogens may provide protection against severe COVID-19. The tuberculosis vaccine Baccilus Calmette-Guérin (BCG) has been studied in this context, as it is known to generally boost the innate immune response and reduce respiratory tract infections (9), with some studies finding a relation between BCG national vaccination programs and COVID-19 mortality rates (10), although there have also been studies that have discarded the protective effects of BCG vaccinations (11). Several other vaccines may ready the immune response with partial specificity, and thereby produce protective effects (12–14). Indeed, there appear to be sequence similarities between proteins in pathogens targeted by common vaccines and SARS-CoV-2 (14–16); e.g. combination vaccines for the infectious diseases diphtheria, tetanus, and pertussis (DTP vaccine) containing cross-reactive epitopes with SARS-CoV-2 (17, 18). Additionally, a team of computer scientists applied an artificial intelligence algorithm in an exploratory fashion to millions of biomedical publications, curated assay databases, and protein databanks. Based on the uncovered associations, they generated a hypothesis that tetanus vaccinations may be linked to COVID-19 severity (19). More directly, one recent study found a high correlation between SARS-CoV-2 molecular response and DTP vaccine proteins. In this same study, severe disease outcomes were reduced among DTP vaccinated COVID-19 patients (20). Together, these clues, coming independently from researchers with distinct backgrounds, provide an intriguing possibility that DTP vaccinations may explain part of the clinical variability of COVID-19 by modulating the immune response.

In most countries, including the United Kingdom (UK), citizens are now vaccinated against tetanus early in life, often together with diphtheria and pertussis or polio (21). Further, five doses of tetanus and diphtheria booster shots later in life are recommended as part of the UK immunization program, as well as shots after incurring wounds or when traveling abroad (22, 23). There is however considerable differences in coverage and adherence between countries and population groups; in the UK, the majority of adult individuals do not carry protective antibodies for tetanus nor diphtheria, particularly the elderly (22, 24). It has also been noted that general population vaccination coverages correlate negatively with COVID-19 mortality rates (13, 19). While claims of causality should be avoided based on such data, cross-reactive immunity could contribute to the greater protection seen for younger individuals, as these have often been recently vaccinated.

Here, we set out to test the hypothesis that DTP vaccinations explain interindividual variation in COVID-19 test results. For this, we used vaccination records from the last ten years of individuals from the large population cohort UK Biobank (UKB), combined with data on COVID-19 testing and related hospitalization, contrasting people vaccinated for diphtheria, tetanus or pertussis *versus* those vaccinated only for other pathogens.

## Materials And Methods

### Study Design

We used data from participants of the UKB population cohort. The composition, set-up, and data gathering protocols of the UKB have been extensively described elsewhere (25). UKB has received ethics approval from the National Health Service National Research Ethics Service (ref 11/NW/0382). For this study, we made use of general practitioner (GP) records that had been linked to only a subset of the UKB participants; given the incomplete linking of these records, i.e. missingness, we selected only participants that had at least one vaccination listed in the GP records in the last ten years, as well as complete information on all covariates. The final sample consisted of 103,049 participants who had a vaccination record in the last ten years and complete covariates.

### Immunization Records

Descriptions of the UK immunization program and vaccination recommendations can be found at https://www.gov.uk/government/publications/immunisation-schedule-the-green-book-chapter-11. We obtained the GP clinical event records linked to the UKB data, downloaded in March 2021. From this, we extracted vaccination information for each participant, which was used to classify them on whether they had received vaccination for tetanus, diphtheria, pertussis, or any other vaccine (influenza, measles, hepatitis, etc.). We upheld strict classifications, only including entries with unambiguous descriptions of specific vaccine administrations. Please see the Supplemental Materials and Methods for an overview of the search terms that were used to query these records in order to determine the classifications. We used the provided registration dates for the immunizations to only include vaccinations that took place in the last ten years, as the effectiveness of many vaccinations wane over time, which is why booster shots are often recommended every ten years (26). Further, this restricts the analyses to the likely more reliable recent registration records. We additionally identified 96 individuals with a BCG vaccination and excluded these, given the substantial amount of literature on a possible relation with COVID-19 severity.

### COVID-19 Confirmed Case

The COVID-19 test results were downloaded from the UKB data repository in April 2021. At that time, the data contained information on 157,884 tests on 77,222 unique individuals. Given multiple tests for many of the individuals, we selected first the most severe, and then the latest positive test, if available. We further added any individual that had COVID-19 registered as cause of death (code U07). Participants with a positive COVID-19 test result, or COVID-19 cause of death are defined as “COVID-19 confirmed cases”.

### COVID-19 Confirmed Severe Case

In accordance with UKB-issued guidance on the analysis of COVID-19 data, we used the inpatient status as indicated in the ‘origin’ field as a proxy for severity. Inpatient status was assigned if the specimen was taken by an emergency care provider, from an inpatient location, or resulting from a hospital-acquired infection. Those individuals with a severe origin or death due to COVID-19 are defined as “COVID-19 confirmed severe cases”.

### Covariates

Differences in registered vaccination, as well as access to health care and lifestyle (e.g., travel and related exposure to pathogens), may relate to socio-economic status (SES), which in turn has been coupled to COVID-19 risk and outcome (27, 28). We therefore included the Townsend deprivation index as a covariate in our analyses, as previously described (29). We flipped the sign of this measure so that higher scores can be interpreted as higher SES.

We additionally covaried for the history of respiratory disease diagnoses based on the International Classification of Diseases v10, codes starting with a ‘J’ (yes/no, n=21934/81115), to lower the potential influence of pre-existing differences in respiratory difficulties between the vaccine groups.

### Statistical Analysis

Differences in demographics between groups were tested through t-tests and chi-square tests, where appropriate.

For the main analyses, we made three sets of cross-sectional comparisons through logistic regression. We compared individuals with, *versus* without, a specific vaccination on 1) whether they had been tested for COVID-19 (yes/no), 2) on whether they are a confirmed COVID-19 case (yes/no), and 3) whether they are a confirmed severe case (yes/no). All analyses were corrected for ‘age’, ‘sex’, ‘SES’, and ‘respiratory disease’.

For each of the three sets of analyses we compared individuals that had been vaccinated for tetanus, diphtheria or pertussis in the last ten years to those that did not receive that specific vaccine or any combination with that vaccine included. For instance, we compared participants that received a tetanus vaccine (either by itself or in combination with other vaccines) to all participants with available vaccination information but no records of tetanus vaccination. Vaccinations are very often combined; we were therefore unable to make exclusive groups, i.e. comparing people that only received one of the vaccines to those that had not. For instance, there were only eight people that had received a diphtheria vaccination and not a tetanus vaccination.

As we used three predictors (diphtheria, tetanus and pertussis vaccination status) and carried out three main tests (testing, outcome, and severity), we set the Bonferroni-corrected significance level to alpha=.05/9=.006 for the cross-sectional analysis.

As a supplementary analysis, we included a step-wise approach, where the proportion of COVID-19 cases was studied with the same methodology but only among those participants that had been tested; and proportion of severe cases only among those that were a confirmed COVID-19 case.

We followed up on the main findings with survival analysis, through Cox proportional hazards models, checking whether individuals with COVID-19, and a vaccination record of diphtheria and/or tetanus had lower or higher risk of developing a severe case of COVID-19 relative to those without a record of diphtheria nor tetanus. All participants with a confirmed COVID-19 diagnostic (n=2,692) and complete covariates were used in the analysis.

The registration of a severe COVID-19 case (n=1,094) was registered as an “event”, and the registration of a non-severe COVID-19 case or loss of follow up as “censoring”. We censored the registration of a non-severe positive COVID-19 case, assuming that people who overcome COVID-19 with mild symptoms are not at risk to develop severe COVID-19 in the future. The survival time was calculated as the number of days from the start of the pandemic (set to March 1^st^, 2020) to the date of the event, censoring, or loss of follow up (set to April 1^st^, 2021).

Survival analyses were corrected for age, sex, SES, and presence of respiratory diseases. For these analyses we first removed those individuals who died during the study period (n=91), as we did not have the date of death available. Subsequently, we analyzed the survival probability among those participants with a positive COVID-19 test (n=2,692).

All analyses were run in R v4.0. Plots were made through the ggplot2 package (30). All pre-processing and analysis scripts are available *via* https://github.com/JenniferMosa/COVID_DTP.

## Results

### Sample Composition and Demographics

The mean age of the 103,048 participants was 71.46 years (SD=6.92) in March 2020; 54.20% of the sample was female. A total of 13,664 participants underwent a COVID-19 test, 2,783 participants had a confirmed diagnosis of COVID-19, and 1,185 participants were either confirmed to have a case of COVID-19 in a hospital setting or died due to COVID-19, which are both considered a proxy for being a confirmed severe COVID-19 case. **Table 1** shows the number of participants with a vaccination record, as well as the number of participants tested, those with a positive diagnosis of COVID-19, and those with a severe COVID-19 case, stratified by each vaccination of interest.

**Table 1:** Number of individuals per vaccination group for each of the study outcomes.

	Vaccination		Undergoing COVID-19 test		Confirmed COVID-19		Confirmed severe COVID-19	
	Yes	No	Yes	No	Yes	No	Yes	No
Diphtheria	5,798	97,251	685	5113	134	5664	30	5768
Tetanus	6,285	96,764	747	5538	150	6135	36	6249
Pertussis	358	102,691	27	331	8	350	4	354

Those participants tested for COVID-19 (n=13,664; 13.3%) were significantly older (+0.2 years, p=8.8*10^-4^), more often male (+2.8%, p=2.8*10^-9^), with a lower SES (-0.23, p=1.7*10^-16^) and higher prevalence of respiratory diseases (+7.7%, p= 8.2*10^-93^) than those not tested. Among those tested, participants testing positive (n= 2,783; 20.4%) were younger (-2.5 years, p=2.6*10^-50^), with lower SES -0.60, p=2.8*10^-19^), and higher prevalence of respiratory diseases (2.7%, p=5.1*10^-3^) than those testing negative, with no differences in sex distribution (p=.59). Among tested positive, those with a severe case of COVID-19 (n=1,185; 42.6%) were significantly older (+3.6 years, p=3.7*10^-32^), more often male (11.2%, p=5.8*10^-9^), had a lower SES (-0.31, p=.01), and higher prevalence of respiratory diseases (5.67%, p=1.2*10^-3^) than those with non-severe cases. **Table S1** shows the demographic characteristics of the sample stratified by vaccination group; those that received any of the DTP vaccinations in the last ten years were more often female, had lower prevalence of respiratory diseases, and were on average younger, and with higher SES than those without these vaccinations.

### Vaccination Record for Diphtheria And Tetanus Is Associated With Lower Odds of COVID-19 Hospitalization

**Figure 1:**
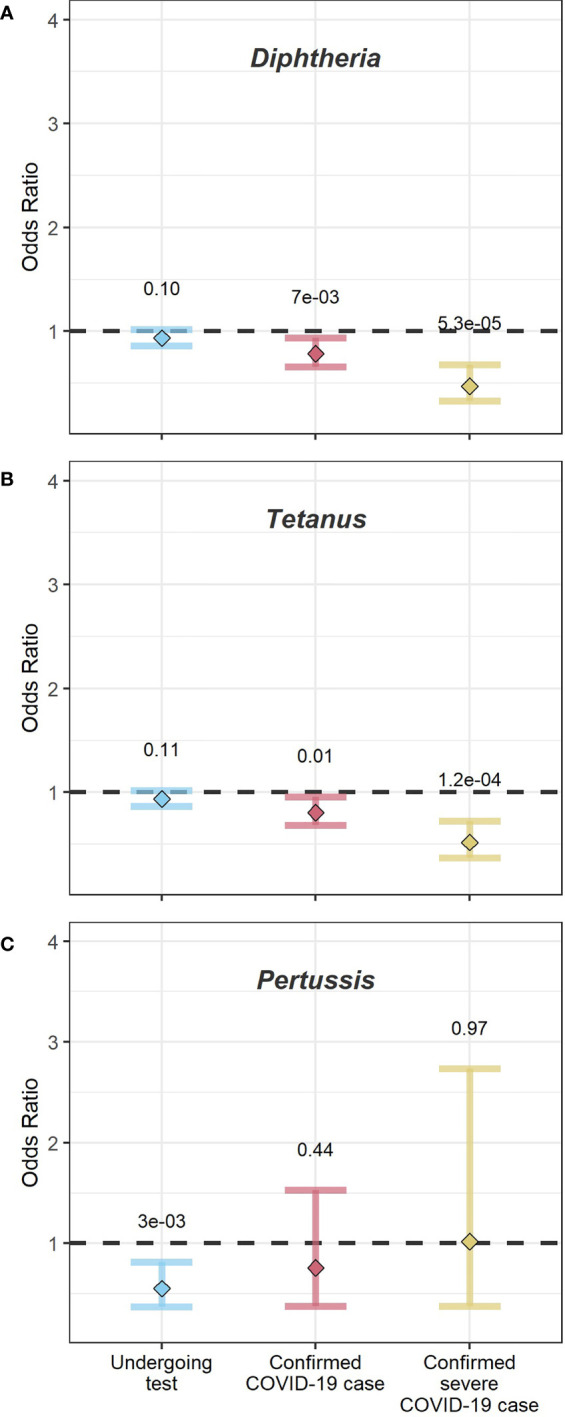
Dot-and-whisker plots from logistic regression analysis across the full sample. The y-axis shows the odds ratios. The x-axis shows the three logistic regression analysis performed (1): undergoing a COVID-19 test (left, blue) (2), being a confirmed COVID-19 case (middle, red), and (3) being a confirmed severe COVID-19 case (yellow, right). Dots indicate the mean odds ratio, and whiskers the 95% confidence intervals. The numbers above the whiskers show the p-value for each analysis. Panel **(A)** shows the outcome for participants vaccinated for diphtheria, relative to those with no record of a diphtheria vaccination, **(B)** shows the outcome for participants vaccinated for tetanus relative to those with no record of tetanus vaccination; and **(C)** shows the outcome for participants vaccinated for pertussis relative to those with no record of vaccination for pertussis.

Comparing individuals with and without a history of diphtheria vaccinations, we found no significant differences in the odds of being tested for COVID-19 (odds ratio (OR) [confidence interval (CI)]=0.93[0.86-1.01]), nor to be a confirmed COVID-19 case (OR[CI]=0.78[0.66-0.94]). However, those vaccinated for diphtheria were less likely to suffer from a severe COVID-19 case than those only vaccinated for other pathogens (OR[CI]=0.47[0.33-0.68]). See **Figure 1A**.

The findings for tetanus vaccination history were similar to those for diphtheria, as shown in **Figure 1B**. Individuals receiving a tetanus vaccination in the last ten years did not differ in odds of getting tested (OR[CI]=0.94[0.87-1.01] or being a confirmed COVID-19 case (OR[CI]=0.81[0.68-0.95]) compared to those without this vaccination. As with diphtheria, they were half as likely to have a severe case (OR[CI]=0.52[0.37-0.72]) than those without this vaccination.

We found that individuals with pertussis vaccinations were less likely to undergo a COVID-19 test (OR[CI]=0.55[0.37- 0.82]), while there were no significant differences in the likelihood to be a COVID-19 case (OR[CI]=0.76[0.37-1.53]) or to be a severe COVID-19 case (OR[CI] 1.02[0.38-2.73]), see **Figure 1C**. We note the small sample sizes for the analyses of pertussis vaccinations. Sensitivity analyses replicated the findings for diphtheria, tetanus, and pertussis through a step-wise approach: Using logistic regression we explored the probability of being a confirmed COVID-19 case only among those people tested; and the probability of being a severe COVID-19 case only among confirmed COVID-19 cases. The observed results pattern was the same as for the analyses over the entire sample (see **Figure S1**).

### Confirmed COVID-19 Cases Vaccinated for Diphtheria and/or Tetanus Are at Lower Risk of Developing Severe Symptoms

Given the findings of significant associations of diphtheria and tetanus vaccinations with the likelihood of being a severe COVID-19 case, we performed follow-up survival analyses through Cox regression among all participants with a confirmed diagnostic of COVID-19, available date of event, and complete covariates (n=2,692).

**Figure 2:**
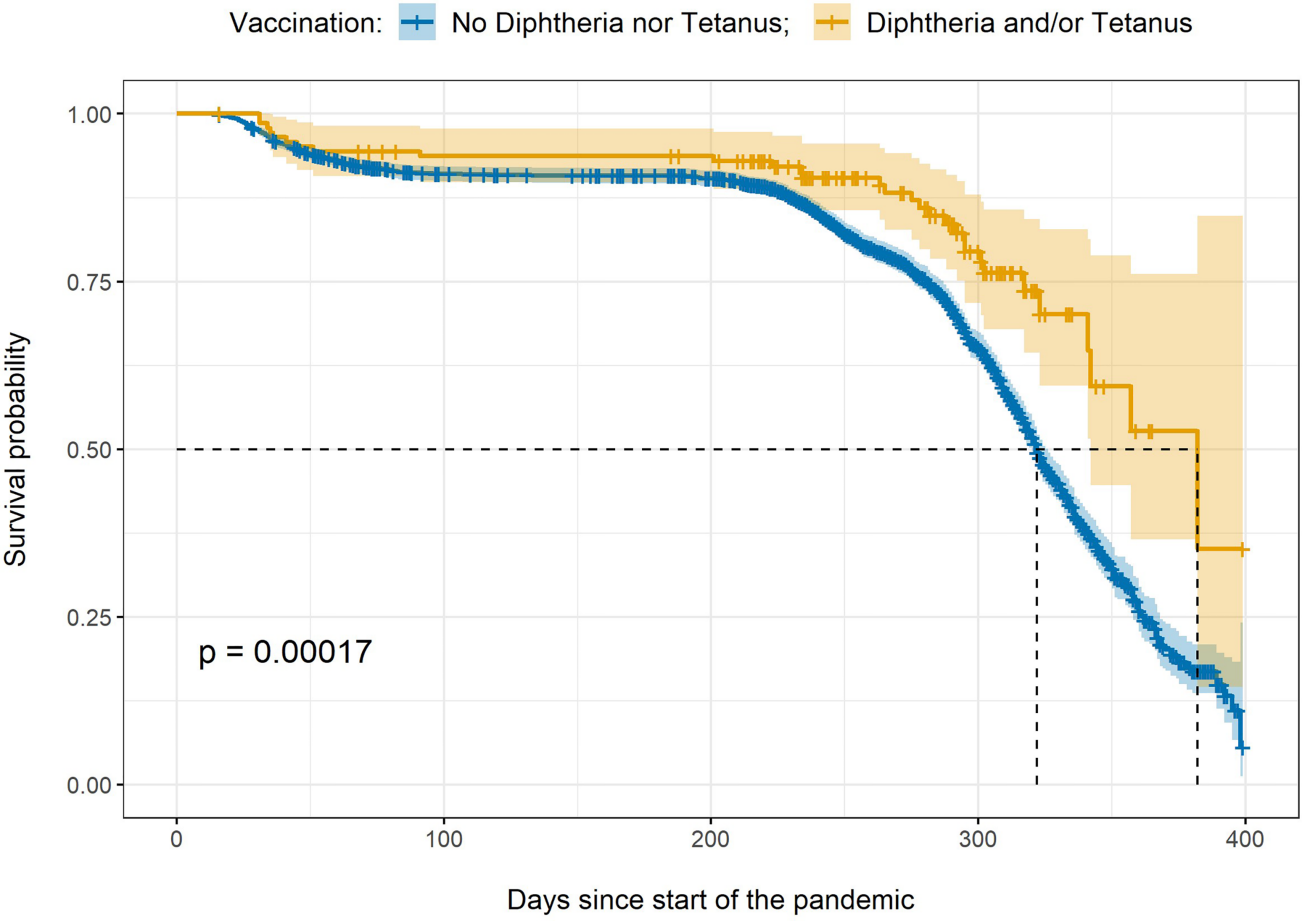
Kaplan Meier curve. The figure shows the survival probability over time and confidence intervals for two groups: those participants with a vaccination record of diphtheria and/or tetanus (in yellow) *versus* those participants with no diphtheria or tetanus vaccinations in their records (in blue). The x-axis indicates the number of days since the start of the pandemic in the UK, the y-axis indicates the survival probability. The dotted lines indicate the median survival time for each group. p = p-value.

We compared participants with a record of diphtheria and/or tetanus vaccinations (n=144) to those with no recorded diphtheria nor tetanus vaccination (n=2,548). **Figure 2** shows the Kaplan Mayer curves for both groups. At the time of analysis 1,094 participants had developed a severe case of COVID-19. The median survival time was 382 days for those vaccinated with diphtheria and/or tetanus, and 322 days for those with no diphtheria or tetanus in their vaccination records.

Relative to those with no diphtheria nor tetanus vaccinations, participants with a vaccination record of diphtheria and/or tetanus were almost half as likely to develop a severe case after correcting for the nuisance variables (hazard ratio [CI]=0.64 [0.44-0.92]; p-value=0.017).

## Discussion

Making use of a large-scale elderly population sample, we found that individuals with a diphtheria or tetanus vaccination in the last ten years were half as likely to suffer from severe COVID-19 than people without these vaccinations. Our follow-up survival analyses further showed that, among those tested positive, people with diphtheria and/or tetanus vaccinations had a lower risk of developing a severe case.

In line with our *a priori* hypothesis, and replicating recent findings (20), our results indicate that DTP vaccinations explain variation in the clinical profile of individuals with exposure to the SARS-CoV-2 virus. Our findings suggest that particularly diphtheria and/or tetanus vaccinations may protect against the development of severe COVID-19 symptoms. A possible mechanism for this would be that these vaccines instill cross-reactive immunity i.e., that they ready the immune response for a SARS-CoV-2 infection, perhaps through protein sequence similarities between the pathogens, as previously indicated (17). Reche further found that no other available vaccine elicits protective cross-reactive immunity against COVID-19 (18). DTP’s possible cross-reactivity is additionally supported by one recent study showing a strong correlation between SARS-CoV-2 response and DTP vaccine proteins (20). The potential protective effects were most evident in the analyses of COVID-19 severity, yet they may also explain more subtle differences in testing and test outcomes between those with and without these vaccinations by suppressing mild symptoms, thereby influencing who shows up for testing.

We emphasize that these study outcomes do not allow for any claims of causality. It is possible that the reported associations are driven by an uninvestigated third factor, that distinguishes those with and without registered DTP vaccinations in the last ten years. This could be e.g., lifestyle, international travel, or access to health care, beyond what was captured by the age, sex, respiratory diseases and SES covariates. We further note that the vaccination records employed in this study may be incomplete to an unknown extent i.e., vaccinations may not have been recorded correctly or completely. We therefore chose to only compare individuals that had registration of specific vaccinations in the last ten years, thereby avoiding that people with missing data introduce biases in the analyses and minimizing the likelihood that these factors drive the results.

It is likely that the immunity conferred by vaccines wanes over time, and tetanus and diphtheria booster shots have been recommended every decade or when traveling (26), although adherence to these recommendations in Europe is low (22). Additionally, routine immunization for tetanus was introduced in the UK only in 1961, meaning the majority of individuals in the UKB have not completed their primary course (21). Such differences in early vaccination history, in addition to interindividual variation in immune system responses, may further explain differences in COVID-19 clinical profiles. It would be particularly valuable for research investigating the potential mechanistic link between vaccinations and COVID-19 response to use more direct, continuous measures of current immune status than the proxy measure of vaccination history employed here.

We were unable to identify whether either the diphtheria or tetanus vaccination drives the results, as too few individuals received exclusively one of the two vaccines. Pertussis has been shown to contribute little to the cross-reactive immunity instilled for SARS-CoV-2 by the DTP vaccinations (18), which fits with our findings, although the small number of observations should be noted. Further, while we excluded individuals with the BCG vaccine based on its suspected relation with COVID-19 severity, some additional vaccines that were contrasted to the DTP vaccines, e.g. Measles or Hepatitis B (13, 16, 20), can potentially have protective effects on COVID-19 symptom presentation as well, thereby obscuring even greater effects of tetanus and/or diphtheria vaccination. It will therefore be important to explore the potential effects of individual vaccines in greater detail, and with more controlled comparison groups.

To conclude, our findings add to literature that specific common vaccines, widely administered to the general population, are associated with severity of COVID-19. These results provide suggestive evidence in favor of what has been previously indicated (17). We do not suggest in any way that these vaccines can be seen as substitutes for vaccines developed for COVID-19. However, our findings may still have significant practical implications, as these vaccinations could explain some of the interindividual variation in the response to COVID-19. Based on these promising results, we call for further research into these associations, ideally making use of direct measures of immune activation and well-matched control groups.

## Data Availability Statement 

This study used UKB population cohort data (https://www.ukbiobank.ac.uk/) under accession number 55392. Dataset is available upon formal request at https://www.ukbiobank.ac.uk/enable-your-research/apply-for-access. All preprocessing and analysis scripts are available via https://github.com/JenniferMosa/COVID_DTP.

## Ethics Statement

The studies involving human participants were reviewed and approved by National Health Service National Research Ethics Service (ref 11/NW/0382). The patients/participants provided their written informed consent to participate in this study.

## Author Contributions

JM-S and DM conceived the study. JM-S and DM pre-processed the data. JM-S performed all analyses, with conceptual input from DM. All authors contributed to interpretation of results. JM-S and DM drafted the manuscript. All authors contributed to the article and approved the submitted version.

## Funding

The authors were funded by the Research Council of Norway (276082, 213837, 223273, 204966/F20, 229129, 249795/F20, 225989, 248778, 249795, 298646, 300767), the South-Eastern Norway Regional Health Authority (2013–123, 2014–097, 2015–073, 2016–064, 2017–004), Stiftelsen Kristian Gerhard Jebsen (SKGJ-Med-008), The European Research Council (ERC) under the European Union’s Horizon 2020 research and innovation programme (ERC Starting Grant, Grant agreement No. 802998) and National Institutes of Health (R01MH100351, R01GM104400).

## Conflict of Interest

OA has received speaker’s honorarium from Lundbeck, and is a consultant to HealthLytix. JP-E has received CME-speaker honoraria from Lundbeck, Angelini, Neuraxpharm, and Janssen, all unrelated to the current work.

The remaining authors declare that the research was conducted in the absence of any commercial or financial relationships that could be construed as a potential conflict of interest.

## Publisher’s Note

All claims expressed in this article are solely those of the authors and do not necessarily represent those of their affiliated organizations, or those of the publisher, the editors and the reviewers. Any product that may be evaluated in this article, or claim that may be made by its manufacturer, is not guaranteed or endorsed by the publisher.
