# Supplementary material for: Diphtheria And Tetanus Vaccination History Is Associated With Lower Odds of COVID-19 Hospitalization
Source: Front Immunol. 2021 Oct 7;12:749264. doi: 10.3389/fimmu.2021.749264 (PMC8529993; doi:10.3389/fimmu.2021.749264)
Supplement: Supplementary file 1 [file DataSheet_1.docx]

Supplementary Material

# Supplementary Data

*Search terms for immunization records*

We queried the general practitioner records to identify which individuals had undergone any vaccinations in the last ten years, followed by grouping individuals based on whether they had received a tetanus, diphtheria or pertussis vaccination. For this, we performed a search for any matches of the strings ‘vacc’ or ‘immuni’ in these records, followed by searches for matches with the vaccine names or common abbreviations, and then manually checking the resulting lists of matched descriptions. From these, we excluded any description containing keywords such as ‘declined’, ‘did not attend’, ‘no consent’, etc. The generated lists of included and excluded descriptions that capture a vaccination event, per vaccine group, was then triple-checked by two authors (J.M.S. and D.v.d.M.). These lists are included as supplementary files. Scripts used for this process can be found at <https://github.com/JenniferMosa/COVID_DTP> > “01_DTP_PrepareDF.R”.

# Supplementary Figures and Tables

**Table S1. Demographics of the sample, split by whether individuals have received a specific vaccine (yes/no).** Standard deviation is indicated behind the point estimates for age and socioeconomic status (SES). The test statistic is a t-value for age and SES and a chi-squared value for sex and history of respiratory diseases. Behind the test statistic we indicate the p-value between parentheses.

| Vaccine | | Age  (mean±SD) | Sex  (% female) | SES  (mean±SD) | Respiratory  (% yes) |
| --- | --- | --- | --- | --- | --- |
| Diphtheria | Yes | 68.23 ± 7.72 | 57.16% | 1.80 ± 2.76 | 12.63% |
|  | No | 71.65 ± 6.82 | 54.02% | 1.36 ± 3.04 | 21.80% |
|  | Test (p) | 32.95 (5.5e-220) | 21.70 (3.2e-06) | -11.65 (4.4e-31) | 275.00 (9.2e-62) |
| Tetanus | Yes | 68.22 ± 7.74 | 56.95% | 1.76 ± 2.79 | 13.08% |
|  | No | 71.67 ± 6.80 | 54.02% | 1.36 ± 3.04 | 21.82% |
|  | T (p) | 34.42 (8.1e-240) | 20.37 (6.4e-06) | -10.71 (1.4e-26) | 269.03 (1.8e-60) |
| Pertussis | Yes | 68.69 ± 7.48 | 56.70% | 1.54 ± 3.00 | 17.32% |
|  | No | 71.47 ± 6.91 | 54.19% | 1.39 ± 3.03 | 21.30% |
|  | T (p) | 7.01 (1.2e-11) | 0.91 (3.4e-01) | -0.97 (3.3e-01) | 3.37 (6.6e-02) |

**
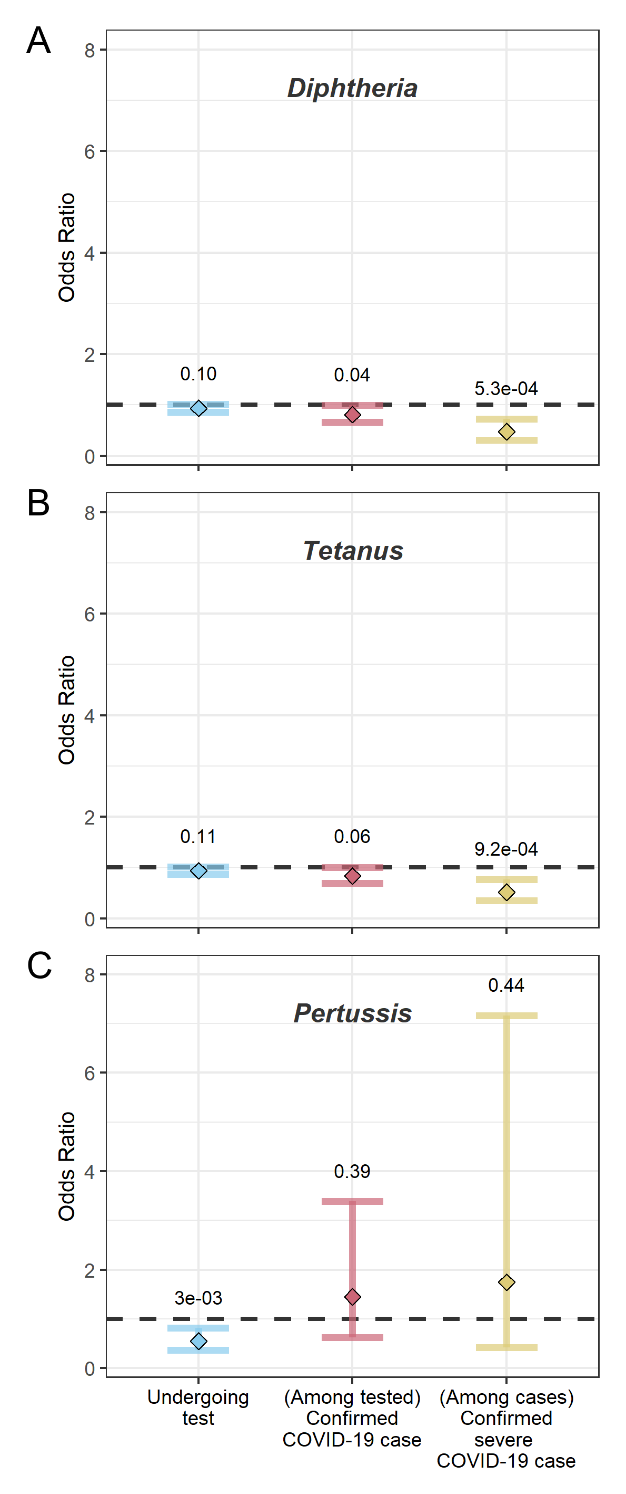
**

**Figure S1: Dot-and-whisker plots from logistic regression analysis: a sensitivity analysis with a stepwise approach.** The y-axis shows the odds ratios. The x-axis shows the three logistic regression analysis performed: (1) undergoing a COVID-19 test (left, blue; this analysis uses the same sample as the main analysis, and hence the results are identical), (2) among tested, being a confirmed COVID-19 case (middle, red), and (3) among confirmed COVID-19 cases, being a severe case (yellow, right). Dots indicate the mean odds ratio, and whiskers the 95% confidence intervals. The numbers above the whiskers show the p-value for each analysis. Panel **(A)** shows the outcome for participants vaccinated for diphtheria, relative to those with no record of a diphtheria vaccination, **(B)** shows the outcome for participants vaccinated for tetanus relative to those with no record of tetanus vaccination; and **(C)** shows the outcome for participants vaccinated for pertussis relative to those with no record of vaccination for pertussis.
